# The Principle and Development of Optical Maskless Lithography Based Digital Micromirror Device (DMD)

**DOI:** 10.3390/mi16121356

**Published:** 2025-11-29

**Authors:** Xianjie Li, Guodong Cui, Guili Xu

**Affiliations:** 1 College of Automation Engineering, Nanjing University of Aeronautics and Astronautics, Nanjing 21106, China; jacklee@ysphotech.com; 2 Jiangsu Yingsu Integrated Circuit Equipment Co., Ltd., Xuzhou 221300, China

**Keywords:** lithography tool, maskless optical lithography system, digital micromirror device (DMD), point array, oblique scanning and stepping

## Abstract

A comprehensive review of the DMD-based optical lithography system has been conducted. The essence of the point-array with an oblique-scanning and stepping operation principle has been systematically analyzed, which will serve as the core driving force for its development and application. Similar to conventional lithography, the system development has been presented from the aspects of critical dimension (CD) resolution, overlay accuracy, and throughput. With the unique characterizations of the digital virtue mask, achievements are summarized from integrated circuit (IC) manufacturing to various micro-scale fabrication processes.

## 1. Introduction

In the integrated circuit (IC) industry, Optical masked lithography is the core process for the manufacturing of microelectronic chips and modules. A conventional lithography system includes a light source, a mask, and an exposure setup. A photomask is used to define and pattern a thin layer of photosensitive polymer, known as photoresist (PR), that is spin-coated onto a substrate. During the development process, the patterned PR microstructures can be formed after chemical etching on the substrate [1].

Due to the ever-increasing mask cost and long delivery period, maskless lithography provides an attractive alternative to mask-based lithography [2]. This approach not only saves mask costs but also provides the ability to make real-time adjustments and offers greater flexibility, making it an increasingly attractive technique in both the semiconductor industry and broader micro-fabrication fields [3]. Among emerging maskless techniques, two primary approaches have been developed: electron beam lithography (EBL) [4], and optical maskless lithography, such as Interference lithography (IL) [5], zone-plate-array lithography (ZPAL) [6,7], and spatial light modulator (SLM)-based maskless lithography (ML) [8,9].

DMD-based optical maskless lithography systems leverage Texas Instruments’ Digital Light Processing (DLP) technology—featuring high mirror switching rates, low manufacturing costs, and broad process compatibility—to balance throughput, flexibility, and cost-effectiveness [10]. Digital light processing (DLP) was developed by Texas Instruments Inc. (TI) and is now dominantly used for front projectors, digital cinema projection, 3D printing, etc. [11]. The key element in DLP is the digital micromirror device (DMD), which contains a matrix of microscopically small mirrors. Each micromirror can be controlled independently to reflect light, enabling the DMD to digitally form an arbitrary optical image. For photolithography, a DMD works with a UV light source and a projection lens system to form a controllable, adjustable UV image on a PR layer. DMD-based lithography creates a virtual image mask on a digital light processing chip, irradiates the grayscale image light source, and then adjusts the exposure time to selectively generate the exposure pattern by projecting the light onto the exposure surface through a micromirror [12]. For large-area UV patterning, the sample is moved by a servo-controlled stage, with movement synchronized with DMD image updates [13].

Now, under the advantages of high resolution, high production efficiency, and low cost, the DMD-based optical maskless lithography system has been widely involved in ICs’ manufacturing, complying with the conventional lithography process, spanning MEMS production [14], micro-optical device processing [15,16,17], 3D micro-nano structure processing [18,19,20], graphic transfer of printed circuit board (PCB) [21,22], flat panel displays (FPDs) [23,24], and so forth.

## 2. A Brief History of DMD-Based Maskless Lithography

**Figure 1:**
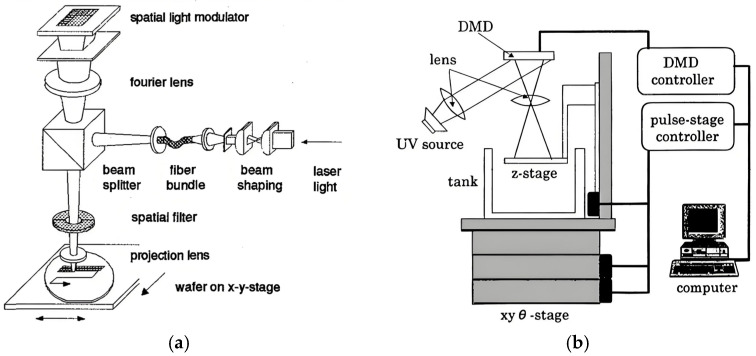
SLM-based maskless lithography: (**a**) programmable SLM [25], (**b**) DMD [8].

As shown in Figure 1, two typical maskless lithography prototypes are presented, which are based on a programmable spatial light modulator, SLM (Figure 1a) and a DMD (Figure 1b). The brief development history of DMD-based maskless lithography is summarized in Table 1.

In 1996, Seltmann et al. unveiled a submicron optical direct-write system using a programmable SLM, achieving 0.6 μm features and one wafer per hour throughput (4-inch)—demonstrating ML’s microfabrication potential [25]. By 2000, Takahi and Setoyama adopted the DMD for UV exposure; its high contrast and low-cost lamp compatibility suited prototyping, though resolution was limited to ~5 μm. This DMD shift later became critical for ML scalability, solving early SLM cost and flexibility issues [8].

- Takahi & Setoyama (2000): First application of DMD in UV exposure [8].

Following their pioneering work, a leap in resolution and system integration emerged, as researchers focused on refining DMD-based setups and expanding ML’s application scope [9,12,36,37,38]. Chan et al. pioneered high-resolution ML systems using Texas Instruments’ SVGA DMDs, combined with low- and high-numerical-aperture (NA) lenses and micro-optics, thereby addressing the resolution limitations of earlier DMD systems. Employing a mercury arc lamp filtered for G-line (435.8 nm), their 2003 work achieved consistent 1.8-μm line/space (L/S) across an 8.47 mm field width, with 1.5-μm L/S observed in optimized conditions. Building on this progress, Yang et al. further enhanced performance by integrating an 848 × 600 microlens and spatial filter array (MLSFA) to reduce noise and shrink UV light spots, enabling sub-16-μm L/S—critical for MEMS and micro-optics [16]. Concurrently, Erdmann et al. (2005) advanced parallel processing capabilities by using a DMD as a switchable mask with a 10:1 demagnification objective, achieving 1.368-μm pixel sizes and processing 800,000 pixels simultaneously [26]. Meanwhile, Sun et al. expanded ML beyond 2D patterning by demonstrating projection micro-stereolithography (PmSL) with DMD, fabricating 3D microstructures (e.g., micro-springs) with 0.6 μm features—opening new avenues for 3D microfabrication [27]. Together, these developments transformed ML from a proof-of-concept into a more versatile tool with improved precision and expanded functionality.

As ML’s technical capabilities grew, the researchers shifted focus to industrial adaptation and precision improvements, tailoring the technology to meet the specific needs of high-volume manufacturing sectors. Kim et al. (2009) led this transition by tailoring DMD-based ML for TFT-LCD manufacturing, a key industry requiring high resolution and uniform patterning [24]. Using 405 nm violet lasers and optical engines with micro-lens arrays (MLAs), their system achieved 2 μm line widths—meeting the strict requirements of display panel production. Building on efforts to enhance manufacturing consistency, Seo and Kim (2010) addressed critical issues of critical dimension (CD) uniformity and throughput with the delta lithography method (DLM), creating honeycomb or square irradiation patterns that reduced line edge roughness (LER)—a key metric for industrial quality control [28]. Ryoo et al. further advanced process understanding by analyzing DMD’s effective reflectance, diffraction efficiency, and line-width control, establishing that a 4-μm diffracted beam spot could achieve practical line widths of ~4.5 μm with 73% exposure efficiency—providing valuable insights for optimizing industrial setups [39]. Additionally, Hansotte et al. (2011) expanded ML’s industrial reach into PCB lithography by introducing gray-level DMD techniques that use larger projected pixels to boost write speed without sacrificing pattern quality [21]. Complementing these manufacturing-focused advancements, Iwasaki et al. (2012) developed a dual-lens DMD system (10×/0.3 NA for wide areas, 100×/0.9 NA for fine patterning), enabling submicron patterns on slanted or deeply etched surfaces—addressing a key challenge for MEMS fabrication on uneven substrates [29,40]. By 2012, ML had evolved from a lab tool into a technology capable of supporting industrial production across multiple sectors.

Post-2014, ML entered a phase of niche and high-volume application expansion, with innovations focusing on reducing costs, improving scalability, and enabling specialized fabrication workflows. Khumpuang et al. (2015) [30] exemplified this trend by integrating DLP-based ML into a “minimal fab” for MOSFET fabrication on half-inch wafers. By operating in an ISO class 4 clean-localized environment instead of a full cleanroom, their system eliminated the need for expensive facility investments while achieving 1-μm gate lengths—making ML accessible for small-scale or research-focused semiconductor fabrication. Building on this drive for accessibility, Diez’s (2016) [31] next-generation Maskless Aligner (MLA) pushed resolution to 1 μm (900 nm pillars) with 50 nm overlay accuracy, using 375 nm and 405 nm lasers and 20 nm stage positioning—accelerating prototyping cycles for microelectronics and MEMS. A key bottleneck for high-volume ML applications—data processing speed—was addressed by Lee et al. in 2018 [13], who used GPU-accelerated rasterization to reduce computation time for continuous DMD frame data. This breakthrough enabled single-overlay exposure for PCBs, further streamlining manufacturing workflows. By the late 2010s, ML had become a flexible solution for both niche research and high-volume production, with cost and efficiency improvements expanding its adoption.

The 2020s have built on these advancements, pushing ML to submicrometer resolution and enabling functional applications in cutting-edge fields like nanophotonics and advanced displays. Kang et al. (2020) [32] set a new benchmark for resolution by using a 200× objective lens to generate 180 nm line widths—half their system’s theoretical resolving power—demonstrating ML’s potential for nanoscale fabrication. Building on this precision, Guo et al. (2021) [33] introduced spatiotemporal modulation (STPL), a technique that coordinates piezoelectric stage movement and DMD pattern generation to reduce the Fresnel zone plate edge sawtooth to 0.3 μm and linewidth error to ±0.1 μm—critical for applications requiring ultra-high pattern fidelity. Choi et al. (2022) [41] balanced speed and precision by optimizing high-speed patterning with pulse exposure and oblique scanning, pushing scanning speeds to DMD damage thresholds while resolving pixelation—addressing a longstanding trade-off between throughput and pattern quality. Most recently, Liu et al. (2023) [34] expanded ML’s nanoscale capabilities by achieving 243 nm narrow gaps in micro-nano structures via femtosecond laser ML, while Syu et al. (2023) [35] demonstrated large-area (160 × 115 mm^2^) 3D patterning for light-guiding plates—solidifying ML’s role in both nanophotonics and large-format display manufacturing. These 2020s innovations have positioned ML as a key enabler of next-generation micro- and nano-fabrication.

## 3. System and Principle of DMD-Based Maskless Lithography

Compared to conventional lithography and other SLM-based maskless lithography(ML), DMD-based lithography could perfectly balance the dilemma of the resolution and throughput to increase process flexibility, reduce the cost of ownership by the essence of digital virtual mask and point array technique [10,11,12,38,42,43].

### 3.1. System Overview

**Figure 2:**
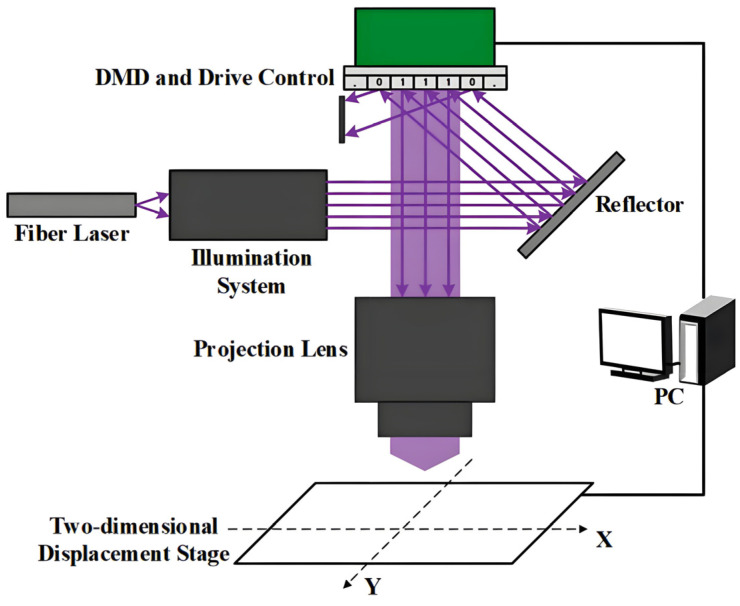
Schematic diagram of a DMD-based scanning lithography system [44].

Generally, a DMD-based lithography system includes at least a light source, illumination, projection, a motion stage, and a DMD subsystem, which the operation software would schedule to complete the exposure procedure. As shown in Figure 2 [44], the schematic diagram of a DMD-based scanning lithography system consists of a fiber laser, illumination system, reflector, DMD with its drive control, projection lens, and a two-dimensional displacement stage. The 405 nm wavelength fiber laser is collimated and homogenized by the illumination system, which includes a homogenizing glass rod and sets of lenses. The parallel light beam emitted by the illumination system is reflected by the reflector onto the DMD. By modulating the digital micromirror array, the DMD directs light selectively into the projection lens, forming the desired dynamic lithography pattern on the surface of the displacement stage. Simultaneously, the displacement stage is driven and controlled in the *X–Y* directions, with its movement speed synchronized to the frame rate of the dynamic pattern displayed on the DMD. By coordinating and synchronizing the on/off operation of the DMD and the movement of the scanning stage, we can achieve arbitrary UV patterning on the photoresist in a massless lithography manner.

### 3.2. Point Array Generation

**Figure 3:**
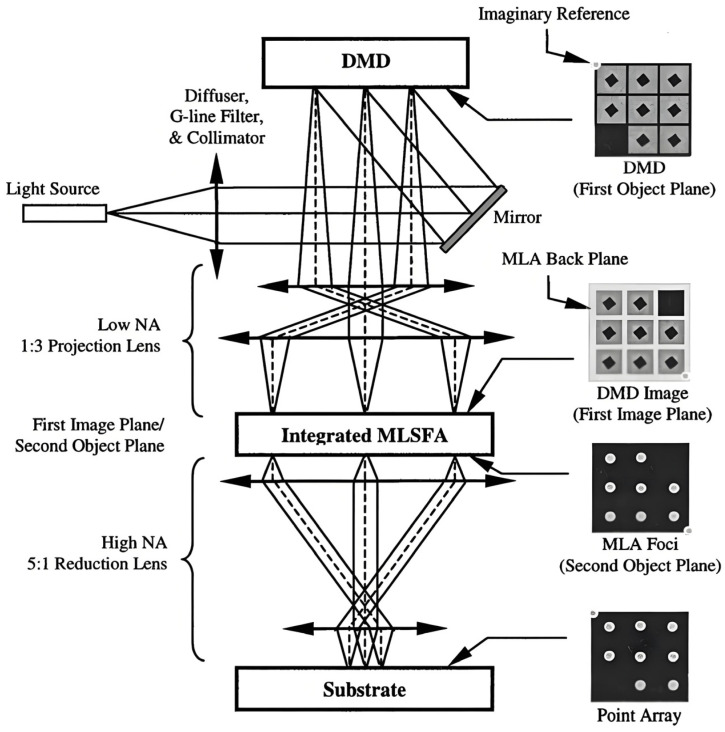
Block diagram of the lithography system [12]. Four diagrams on the right (from top to bottom) describe the appearance of the DMD, the DMD inverted image coplanar with the MLA back plane, the MLA foci coplanar with the spatial filter array, and the point array projected to the substrate. These diagrams are not adjusted for image magnification or reduction.

Figure 3 [12] shows the block diagram of the lithography system to generate an array of points. The expanded and homogenized laser beam is collimated by a series of optical components before it is incident on the DMD surface. The digital micro-mirror device (DMD) operates as an optical shutter for image projection, using a slanting two-dimensional array of tens of thousands of infinitesimal mirrors, made by Texas Instruments Inc., the mirror for each pixel can be tilted by static electricity so that the path of the incident light can be changed (on/off).

The beam is reflected by a mirror, then passes through an aperture and a condenser lens to obtain a uniform plane wave, which is reflected by the DMD. Then the beam will pass through another aperture to cut off the extra light outside the DMD pattern and be reflected by the second mirror to a projection lens set. First, the DMD field image is projected onto the integrated microlens/spatial-filter array (MLSFA) using a low-NA magnifying projection lens. The focal plane of the MLSFA becomes the second object plane. The second lens assembly consists of a high-NA reduction lens that forms a projected UV point array on the substrate.

### 3.3. The Operation Principle

Early DMD lithography used step-and-repeat: exposing statically, then moving the stage. It was slow for large areas due to frequent stage acceleration/deceleration [36,45]. In contrast, DMD scanning lithography—generating stationary patterns while the stage moves continuously via high DMD frame rate—boosts maskless lithography throughput [38].

To enhance patterning resolution and achieve a large patterning area, the projected DMD images are obliquely scanned across the substrate surface at a small tilt angle. This ensures that the light emitted by each individual digital micromirror of the DMD is evenly distributed either horizontally or perpendicularly relative to the scanning direction [35,46,47,48,49,50].

**Figure 4:**
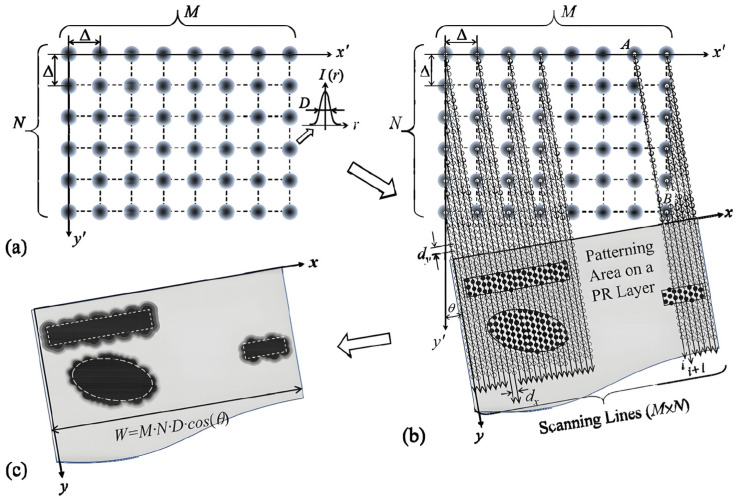
Schematic of oblique scanning and step-strobe light (OS^3^ L) scheme for UV-patterning using a DMD-based lithography system [49]: (**a**) Projected array of UV spots; (**b**,**c**) maskless UV patterning based on point array oblique scanning method.

As shown in Figure 4, A DMD-based scanning-type maskless lithography system with an oblique scanning and step-strobe lighting (OS3 L) scheme is presented, which could enhance the patterning capability together with better quality [46,49].

To perform maskless UV patterning, the array of UV spots is scanned obliquely along the *y*-axis. All of the UV spots have their own scanning lines. Furthermore, the ON/OFF status of each spot can be controlled individually by the digital image sent to the DMD chip. Therefore, UV patterning can be viewed as a stepping UV exposure process with an incremental step size of $d_{y}$ along the scanning lines. During the scanning process, digital image data are sent continuously to the DMD in every scanning step of $d_{y}$ to update the ON/OFF status of the UV spots.

To ensure that the scanning lines are equally spaced, the oblique scanning angle, θ is set as follows:

(1) $$\theta ={tan}^{-1}(1/n),$$

where n is an integer representing the slope of the scanning line, and the spacing between adjacent scanning lines, $d_{x}$, is then given by the following:

(2) $$d_{x}=\frac{⧍\;\cdot \;COS\theta }{n},$$

⧍ is the projected DMD pixel size.

The point-array oblique-scanning method described above produces a well-defined UV intensity profile for each UV spot by eliminating the high spatial-frequency scattered UV light produced by the micromirrors. Furthermore, it achieves an extremely fine pattern resolution since the spacing between scanning lines ($d_{x}$) can be very small, and the stepping distance ($d_{y}$) is precisely controlled by a mechanical scanning stage.

Each selected UV exposure point represents the projection of a specific UV dose onto the designated area surrounding that point. The distribution of the projected UV dose from a single DMD pixel during each step size is calculated as follows:

(3) $$u_{0}=\frac{1}{v}\int_{0}^{\mu d_{y}}I(x,y-s)ds,$$

where I(x,y) is the projected optical power intensity from a single DMD pixel; v is the scanning speed; and μ is the chosen duty cycle for the ON/OFF switching of the UV light within each step size [35,46].

### 3.4. DMD and System Imaging Model

As the heart component, the characterization of the DMD has been extensively studied, which would critically define the performance of the lithography system performance [11,51,52,53]. Following the principle of conventional lithography, an image model with consideration of DMD’s unique optical features is developed to guide the design and optimize the system performance [4,10,13,24,48,54,55,56,57,58,59,60,61,62,63,64,65,66,67,68,69,70,71,72,73,74,75].

#### 3.4.1. DMD and Characterizations

**Figure 5:**
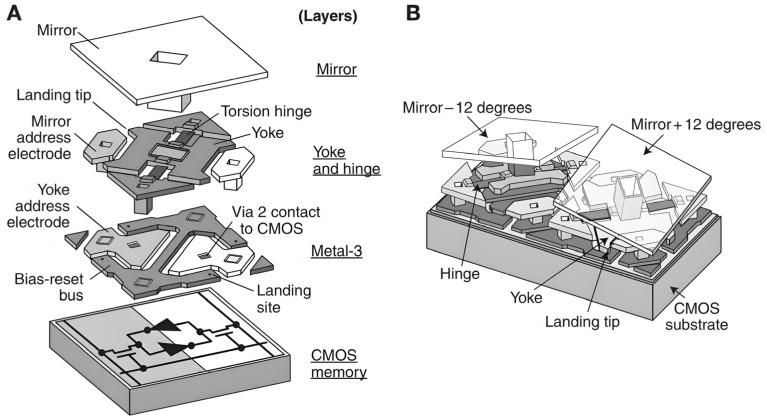
DMD microstructure [51]: (**A**) The DMD is composed of a square mirror on top of a diagonal hinge. A complementary metal-oxide semiconductor (CMOS) memory controls the landing pads that determine the orientation of the mirror. (**B**) The mirrors are mechanically constrained to tilt angles of ±12°. The DMD acts as a binary light switch (Images from Hornbeck 1997 [76]).

The Digital Micromirror Device (DMD) developed by Texas Instruments (TI) is both a micro-electromechanical system (MEMS) and a spatial light modulator (SLM). The DMD consists of hundreds of thousands of moving micromirrors, from 1024 × 768 extended graphics array (XGA), 1400 × 1050 superextended graphics array plus (SXGA+), and 1920 × 1080 high-definition television (HDTV). which are controlled by underlying CMOS electronics. As shown in Figure 5, The device consists of an array of micromirrors, 16 µm × 16 µm square, mounted on a 17 µm pitch and can rotate to either +12° or −12° position along the diagonal direction depending on the binary state of the SRAM cell planted below each mirror, So each individual mirror can acts as an on/off switch by either reflecting light towards the optical system or by reflecting light away from it. With a minimum chip size of 13.7 μm or 10.6 μm, the data transfer speed can reach 25.6 Gb/s (52,550 frames/sec) for the XGA chip and 24,690 frames/sec for the HDTV chip.

**Figure 6:**
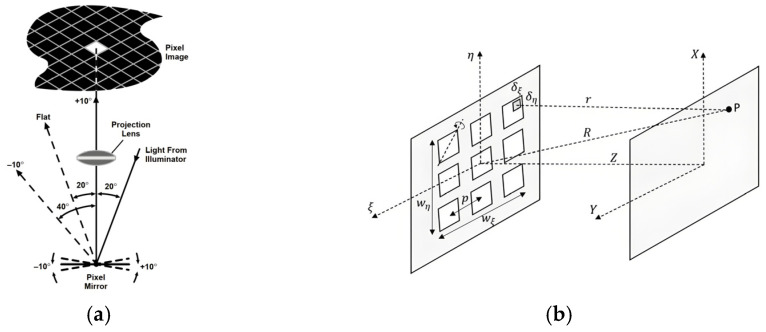
The modulation of light: (**a**) DMD optical switching principle [76], (**b**) Diagram taking into consideration to calculate the diffraction pattern of a two-dimensional array of rotated micromirrors. The aperture array is placed at the (*ξ*, *η*) plane, and the screen target is placed at the (*X*, *Y*) plane. The *Z* axis is shared by both coordinate systems. The planes are parallel to each other, at a normal distance *z*. *δξ* and *δη* represent an arbitrary small area of one square aperture, where a wavefront is emitted and contributes to the diffraction pattern at an arbitrary point *p*, at a distance *r* from the center of the small area (*δξ*, *δη*) and at a distance *R* from the origin of the (*ξ*, *η*) coordinate system [53].

By rotated a θ angle, the light propagation direction could be changed as shown in Figure 6a [53]. Due to the order of pitch size, the Fraunhofer far field distribution is comprehensively analyzed using angular theory. Based on the diagram of Figure 6b, the intensity on the z-plane is given as follows [53]:

(4) $$I(x,\;y)=\frac{A^{2}}{\lambda^{2}z^{2}}{sinc}^{2}[l(\frac{x}{\lambda z}-t_{0})]{sinc}^{2}[l(\frac{y}{\lambda z}-t_{0})]{\left\{\sum_{i,j}e^{-2\pi j[\xi_{ij}\left(\frac{x}{\lambda z}-t_{0}\right)+\eta_{ij}\left(\frac{y}{\lambda z}-t_{0}\right)]}\right\}}^{2},$$

where *l* is the size of the micromirror, equal for both *ξ*, *η* directions; $w_{\xi }$, $w_{\eta }$ are the dimensions of the aperture array in *ξ*, *η* directions; and *p* is the pitch between apertures and $t_{0}$ is defined as $l^{2}sin\theta /\sqrt{2}$ to describe the rotation effect different from planar two-dimensional diffraction.

#### 3.4.2. Imaging Model

In conventional lithography, the Hopkins model is used to describe propagation and imaging for partial coherent fields, as follows [77]:

(5) $$I(f,g)=\iint \iint_{-\infty }^{-\infty }TCC\left(f^{\prime }g^{\prime },f^{\prime \prime }g^{\prime \prime }\right)M^{*}(f^{\prime },g^{\prime })M^{*}(f^{\prime \prime },g^{\prime \prime })df^{\prime }dg^{\prime }df^{\prime \prime }dg^{\prime \prime },$$

(6) $$I\left(x,y\right)=F^{-1}[I(f,g)],$$

where *f* and *g* represent frequency domain coordinates; “*” is complex conjugation; *x* and *y* represent spatial coordinates; *I*(*x*, *y*) and *I*(*f*, *g*) are two-dimensional Fourier transforms of spatial light intensity and spatial light intensity, respectively; *M*(*f*, *g*) is the Fourier transform of the mask *M*(*x*, *y*); and *M*(*F*, *G*) is the Fourier transform of the mask *M*(*x*, *y*). *TCC*(*f*′, *g*′, *f*″, *g*″) is a transmission cross coefficient, a two-dimensional convolution integral independent of the mask pattern, which describes the function of the entire optical imaging system from the illumination source to the image plane.

During the lithography process, aerial imaging *I*(*x*, *y*) in the substrate could be formed as follows:

(7) $$I\left(x,y\right)={\left|F^{-1}[O(u,v)H(u,v)\right|}^{2},$$

where $\;I\left(x,y\right)$ is image intensity on the wafer plane; O(u,v) is the Fourier transform of the object function on the mask plane; H(u,v) is transfer function; F^−1^ is inverse Fourier transform operation; $\left(x,y\right)$ is two-dimensional spatial coordinates; (u,v) is two-dimensional frequency coordinates.

Under the scheme of the oblique scanning along x with a tilt angle θ, as shown in Figure 4, for the DMD-modulated aerial image illuminated with intensity $I_{0}$ could be written as follows:

(8) $$I\left(x,y,t\right)=I_{0}{\left|Re\left[u(x,y)\right]\right|}^{2},$$

where u(x,y) is the intensity distribution with consideration of binary on/off modulation may be written as follows:

(9) $$u\left(x,y\right)=\sum_{m=0}^{M-1}\sum_{n=0}^{N-1}\exists_{mn}\varepsilon^{2}rect\left(\frac{x-mC}{C}\right)rect\left(\frac{y-nC}{C}\right)exp\left\{-i\frac{2\pi }{C}\left[j\left(x-mC\right)+k(y-nC)\right]\right\},$$

where $\exists_{mn}$ is a binary parameter which yields the approval of ON/OFF reflection of each micromirror upon the input pattern; C is the pixel size; and e is the square root of the fill factor [55,57,78].

### 3.5. Exposure Model

The exposure dose E overtime ⧍t_i_ given target pattern *g(x, y)* on the photoresist can be expressed as follows [59,78]:

(10) $$\Delta E_{i}\left(x_{i},y_{i}\right)=\Delta I_{i}\left(x_{i},y_{i}\right)x\Delta t_{i},$$

where

(11) $$\Delta I_{i}\left(x_{i},y_{i}\right)=\iint I_{eff}\;\;\times {\left|\iint U(g_{x}{,g}_{y},\Delta t_{i})H(x_{s}-g_{x},y_{s}\;\;-g_{y})\times exp\left[j2\pi (g_{x}x_{i},g_{y}y_{i})dg_{x}{dg}_{y}\right]\right|}^{2}dx_{s}{dy}_{s},$$

And $I_{eff}$ is the effective light intensity after the pulse width modulation (PWM) modifies the beam intensity, $H(x_{s}-g_{x},y_{s}-g_{y})$ is the transfer function of the optical system, and $U(g_{x}{,g}_{y},\Delta t_{i})$ is the amplitude in the DMD spatial spectrum over time $\Delta t_{i}$.

The total exposure dosage *E* over time $\Delta t_{i}$ can be expressed as follows:

(12) $$E(x,\;y)\;=\;\sum_{i=1}^{n}\Delta E_{i}\left(x_{i},y_{i}\right),$$

where *n* is the repeat number by different element of the DMD determined by oblique scanning angle θ.

A single scan virtual layering (SSVL) exposure model is developed to describe the overlaid scan with multi-pass [78].

To predict the interaction between optical intensity distribution and, the Dill exposure model plays an important role in the conventional optical lithography and has been widely studied and developed [79,80,81,82,83,84,85]. Together with the dose distribution, the target pattern *g(x*, *y)* could be determined by the photonic active compound (PAC) concentration *M(x*, *y)* as follows:

(13) $$M(x,y)\;=\;M_{0}exp[\left(-CE\left(x,y\right)\right],$$

where *M*_0_ and *C* denote the initial concentration of PAC and one of exposure parameters.

### 3.6. Digital Pattern Generation

To generate lithographic patterns, millions of micromirrors in the array must be addressed and adjusted individually and instantaneously. Providing the micromirror controller with a stream of lithographic pattern signals matching the substrate’s relative movement is a non-trivial task. Several pattern generation methods have been presented to increase the robustness and flexibility of the pattern [37,58,86,87,88,89,90,91,92,93,94,95,96].

**Figure 7:**
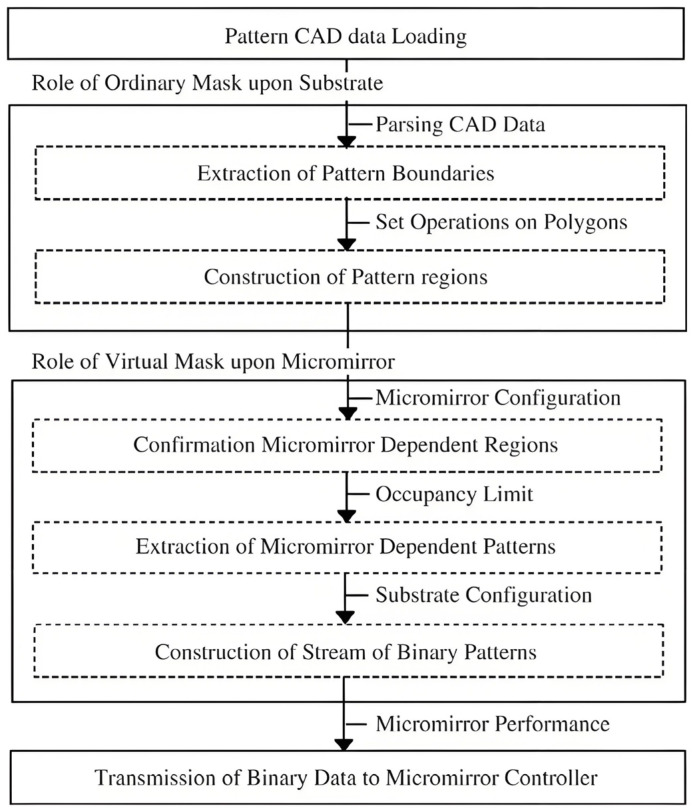
Lithographic pattern generation flow [92].

The flow of the lithographic pattern generation process is shown in Figure 7 [92], which consists of three key procedures. First, the initial procedure focuses on loading CAD data—typically in formats such as Drawing eXchange Format (DXF)—through a data parsing process. Second, the intermediate procedure acts as an ordinary mask for vector pattern generation and involves two core routines: it reconstructs geometric entities with open loops into closed loops to extract pattern boundaries and performs set operations on polygons (based on computational geometry principles) to construct pattern regions. Finally, the third procedure functions as a virtual mask for raster pattern generation, with three associated routines: it confirms micromirror-dependent lithographic pattern regions according to the micromirror configuration, extracts micromirror-dependent patterns to determine binary reflection based on occupancy, and constructs a stream of binary patterns (incorporating binary reflection information for the micromirrors) in line with substrate translation.

A critical step is to discriminate precisely between the approval of the on/off states of the DMD mirror for pattern fidelity. At the same time, the generated data should be compressed to satisfy the requirements of high-speed real-time and large area exposure [80].

## 4. The Recent Progress

Following the development of conventional lithography technology, finer resolution, better overlay accuracy, and higher throughput remain a persistent pursuit.

Maskless Optical Projection Nanolithography (MLOP-NL) overcomes the optical diffraction limit, achieving a minimum feature size of 32 nm (λ/12), enabling the fabrication of multi-scale micro-nano hybrid structures [97]. Because the illumination played a vital role in CD resolution, several schemes have been designed to improve the uniformity and power energy [98,99].

By exposure algorithm and scanning strategy optimization, intra- and inter-field overlay error is improved. The edge sawtooth is smoothed by the pixel collaborative dynamic lure technique [100,101]. Inverse lithography technology (ILT) is used to improve the pattern fidelity [71,102,103]. Using the stage self-calibration, the main source of overlay error has been greatly decreased, and this promotes the system’s stability [104,105,106]. The intra-field registration has been lower to ~1um by image super-pixel precision distortion correction [107,108,109,110]. A multi-layer exposure alignment system for DMD lithography, achieving *x*-direction average alignment accuracy of 1.76 μm and y-direction of 0.63 μm (max error ≤ 3 μm) [111].

With the path planning optimization, the scanning and stepping time is reduced by 30~40% [112,113]. DMD-based array one-shot exposure saved the 3D exposure time by virtue of millions of mirrors, and avoided the stitching effect [18,114,115,116].

The diagnosis and calibration method has been developed to assure the system stability by on-machine real-time illumination monitoring [110,117,118].

To benefit from the performance’s continuous improvement, DMD-based maskless lithography technology has been involved in micro-scale IC manufacturing, and application areas continue to expand. It has achieved <2 um alignment accuracy, 4 um micro-features, and is expected to be below 500 nm, confirming the potential in advanced packaging [119,120,121]. In the PCB industry, low-cost and fine-line exposure lithography has been realized with 2 um resolution and ≤1 um overlay, which would meet advanced PCB manufacturing requirements [122,123,124,125,126,127].

Because of its cost-effectiveness and flexibility, it has been used to fabricate microlens arrays (MLAs) and diffraction optical element (DOE) arrays. Fresenel MLAs, conical MLAs, and high-filled aspherical MLAs have been reported [128,129,130]. Under the experience of MLAS, it is introduced to the fabrication of higher demand DOEs, such as Shack-Hartmann wavefront sensor, Fresnel zone plate, etc. [115,131,132]. Furthermore, with the assistance of brighter intensity femtosecond laser resources, some research in photonic integration has advanced [97,133,134].

Combined with the fine 2D and 3D pattern capabilities, researchers try to construct a 3D microstructure to simulate cell behavior via DMD maskless lithography. The innovative application has been realized in 3D extracellular microenvironments for cells, microchannels with 10 um Z-resolution and rapid protein pattern, etc. [114,135,136,137].

## 5. Conclusions and Outlook

A comprehensive review of the DMD-based optical lithography system has been conducted, with the objective of clarifying its technical mechanism, performance boundaries, and industrial application potential. A central research focus lies in the theoretical and experimental analysis of two core operational principles: (1) the point array generation mechanism, which relies on microlens arrays and spatial filters to form uniform UV point arrays on the photoresist substrate, laying the foundation for high-resolution patterning; (2) the oblique scanning-stepping cooperative operation, where oblique scanning could help subpixel-resolution and stepping stitching enables large-area seamless patterning.

Recent advancements have significantly enhanced the maturity of DMD-based lithography systems, with performance approaching or meeting mid- to low-end microfabrication requirements. In terms of CD resolution, integrating Maskless Optical Projection Nanolithography (MLOP-NL) and inverse lithography technology (ILT) has reduced the minimum feature size to 32 nm (λ/12, λ = 405 nm laser), while mainstream systems stably achieve 180–500 nm resolution. For overlay accuracy, stage self-calibration and DMD pattern distortion correction have controlled intra-field registration error to ~1 μm, and multi-layer alignment accuracy reaches sub-2 μm—critical for IC advanced packaging and MEMS multi-layer structures. In terms of throughput, path planning optimization (e.g., dynamic pixel collaboration) and array one-shot exposure have reduced scanning/stepping time by 30–40%, enabling a single system to achieve ~10 wafers/hour (8-inch) throughput for 2 μm resolution patterns.

Future development of DMD-based lithography will focus on technical breakthroughs and industrialization advancement. Technically, resolution enhancement will explore metamaterial lenses (to reduce spherical aberration) and femtosecond laser-DMD hybrid systems, targeting sub-30 nm CD resolution; overlay precision improvement will integrate multi-sensor fusion (e.g., laser interferometry + machine vision) and deep learning-based real-time calibration to reduce overlay error to ≤500 nm; throughput optimization will adopt large-area DMD arrays (e.g., 4 K/8 K DMD) and GPU-ASIC collaborative data processing to boost throughput to >20 wafers/h (8-inch). For industrialization, standardization (e.g., digital pattern file formats, performance testing protocols) will enhance equipment-substrate compatibility; penetration into emerging fields (nanophotonics, quantum technology) will leverage high customization and low cost for rapid prototyping; and sustainable manufacturing (reduced waste via digital masks) will support flexible electronics (wearable sensors, displays) with on-demand patterning. In conclusion, the technology has evolved from lab-scale to a promising tool, and future efforts will unlock its potential as a critical complement to conventional lithography in diversified micro- and nano-manufacturing.

## Figures and Tables

**Table 1:** Critical stages and representative works of DMD-based maskless lithography.

Critical Stage	Time Period	Representative Work	Comment
Technological Inception	1996–2000	- Seltmann et al. (1996): Submicron optical direct-write system based on programmable SLM [25]. - Takahi & Setoyama (2000): First application of DMD in UV exposure [8].	- Verified ML’s microfabrication potential. - Laid the foundation for DMD-based ML by solving early SLM cost and flexibility issues.
Performance Breakthrough	2003–2005	- Chan et al. (2003): High-resolution ML system with TI’s SVGA DMD [9,12]. - Yang et al. (2003): MLSFA-integrated system for noise reduction [16]. - Erdmann et al. (2005): DMD as switchable mask for parallel processing [26]. - Sun et al. (2005): DMD-based projection micro-stereolithography (PmSL) [27].	- Significantly improved resolution (down to 1.5 μm L/S). - Realized parallel processing and expanded ML to 3D microfabrication.
Industrial Adaptation	2009–2012	- Kim et al. (2009): DMD-based ML for TFT-LCD manufacturing [24]. - Seo & Kim (2010): Delta lithography method (DLM) for CD uniformity [28]. - Hansotte et al. (2011): Gray-level DMD tech for PCB lithography [21]. - Iwasaki et al. (2012): Dual-lens DMD system for uneven substrates [29].	- Adapted ML to high-volume industries (TFT-LCD, PCB, MEMS). - Transformed ML from a lab tool to an industrial-grade technology.
Cost and Efficiency Optimization	2014–2019	- Khumpuang et al. (2015): “Minimal fab” with DLP-based ML [30]. - Diez (2016): Next-generation Maskless Aligner (MLA) [31]. - Lee et al. (2018): GPU-accelerated rasterization for data processing [13].	- Reduced application costs (no full cleanroom required). - Broke through high-volume production bottlenecks, expanding ML’s adoption.
Cutting-Edge Expansion	2020–Present	- Kang et al. (2020): 180 nm line width with 200× objective [32]. - Guo et al. (2021): Spatiotemporal modulation (STPL) for ultra-high fidelity [33]. - Liu et al. (2023): Femtosecond laser ML for 243 nm gaps [34]. - Syu et al. (2023): Large-area 3D patterning for advanced displays [35].	- Pushed ML to submicron/nanoscale resolution. - Expanded ML to cutting-edge fields (nanophotonics, advanced displays).

## Data Availability

No new data were created or analyzed in this study. Data sharing is therefore not applicable to this article.
